# Optimizing Health for Canadian Youth at Clinical High Risk for Psychosis: A Feasibility Study: Optimiser la santé des jeunes Canadiens présentant un risque clinique élevé de psychose : Une étude de faisabilité

**DOI:** 10.1177/07067437261436536

**Published:** 2026-04-07

**Authors:** Yun Lu, Beatrice Todesco, Lisa D. Hawke, Nicole Kozloff, Gillian Strudwick, Michael Kiang, Wei Wang, David Castle, George Foussias, Muhammad Omair Husain

**Affiliations:** 1 7978 Campbell Family Mental Health Research Institute, Centre for Addiction and Mental Health, Toronto, Ontario, Canada; 2 Department of Psychiatry, Temerty Faculty of Medicine, 7938 University of Toronto, Toronto, Ontario, Canada; 3 Institute of Health Policy, Management and Evaluation, 7938 University of Toronto, Toronto, Ontario, Canada; 4 Department of Psychiatry, 7978 University of Tasmania, Tasmania, Australia; 5 Centre for Mental Health Service Innovation, Statewide Mental Health Service, Tasmania, Australia

**Keywords:** psychosis, clinical high risk, psychosocial intervention, OHP-CHR

## Abstract

**Objective:**

We adapted and evaluated a transdiagnostic psychosocial intervention, the Optimal Health Program (OHP), to meet the unique mental health needs of youth at clinical high risk for psychosis (CHR). This study aimed to establish the acceptability of OHP for CHR (OHP-CHR), the feasibility of conducting a confirmatory clinical trial, and the preliminary efficacy of the intervention in improving clinical and functional outcomes.

**Methods:**

In this single-arm clinical trial, youth aged 16–29 years meeting CHR criteria were recruited between September 2023 and August 2024. Nine OHP-CHR sessions were delivered over 12 weeks. Acceptability of the intervention was assessed through session attendance rates and the Client Satisfaction Questionnaire (CSQ-8). Feasibility of conducting a confirmatory clinical trial was informed by recruitment, retention, adherence, intervention fidelity, and completion of the outcome schedule. Psychiatric symptoms, resilience, and functioning were assessed at baseline and after 12 weeks.

**Results:**

Acceptability of OHP-CHR was demonstrated by high session attendance (*n* = 26; 86.7% attended all nine sessions) and participant satisfaction scores (CSQ-8: mean = 28.35, SD = 3.68). Feasibility of conducting a confirmatory clinical trial was supported by strong recruitment over 12 months (*n* = 30; rate of 2–3/month) high retention (*n* = 26; 86.7% at 12 weeks), intervention fidelity, and ease of completing the clinical assessment schedule. Preliminary indicators of clinical efficacy were demonstrated by a decrease in psychiatric symptoms, improvement in resilience, and enhanced functioning.

**Conclusions:**

OHP-CHR is a promising psychosocial intervention co-designed by CHR youth. Our data support the acceptability of the intervention, the feasibility of conducting a confirmatory clinical trial, and preliminary evidence of efficacy in improving clinical and functional outcomes.

Trial is registered at ClinicalTrials.gov NCT05757128.

## Plain Language Summary Title

Testing a well-being and self-efficacy programme for Canadian youth at risk for psychosis

**Plain Language Summary:**

**Objective**

We adapted and tested a mental health programme called the Optimal Health Program (OHP) to better support youth who are at clinical high risk for psychosis (CHR). The goal of this study was to see if the adapted version, called OHP-CHR, was acceptable to young people, whether it would be possible to run a larger clinical trial in the future, and whether the programme could help improve mental health and daily functioning.

**Methods**

We invited youth aged 16 to 29 who met CHR criteria to take part between September 2023 and August 2024. Participants attended nine OHP-CHR sessions over 12 weeks. We looked at how many sessions people attended and how satisfied they were to understand if the programme was acceptable. To see if a larger study would be feasible, we tracked recruitment, retention, session attendance, programme delivery, and completion of assessments. We measured psychiatric symptoms, resilience, and functioning before and after the programme.

**Results**

The programme was well accepted. We successfully recruited 30 participants over 12 months and retained 87% through the end of the study. Most participants (87%) attended all sessions and reported high satisfaction. The programme was delivered as planned, and participants found the assessments easy to complete. After completing OHP-CHR, participants showed fewer symptoms, greater resilience, and better functioning.

**Conclusions**

OHP-CHR is a promising mental health programme developed with input from CHR youth. Our findings show that young people find it acceptable and engaging, and that a larger clinical trial would be feasible.

## Résumé

**Objectif:**

Nous avons adapté et évalué une intervention psychosociale transdiagnostique, l’Optimal Health Program (programme OHP), pour répondre aux besoins uniques en santé mentale des jeunes présentant un risque clinique élevé de psychose. Cette étude visait à établir l’acceptabilité du programme OHP pour les jeunes présentant un risque clinique élevé de psychose, la faisabilité de l’essai clinique de confirmation et l’efficacité préliminaire de l’intervention sur le plan de l’amélioration des résultats cliniques et fonctionnels.

**Méthodes:**

Dans cet essai clinique à un seul groupe, des jeunes de 16 à 29 ans répondant aux critères de risque clinique élevé de psychose ont été recrutés entre septembre 2023 et août 2024. Neuf séances du programme OHP pour les jeunes présentant un risque clinique élevé de psychose ont été offertes sur une période de 12 semaines. L’acceptabilité de l’intervention a été évaluée au moyen des taux de présence aux séances et du questionnaire sur la satisfaction de la clientèle (CSQ-8). La faisabilité de l’essai clinique de confirmation a été estimée à l’aide de donnée sur le recrutement, la rétention, l’observance, la fidélité à l’intervention et la facilité à respecter le calendrier d’évaluations. Les symptômes psychiatriques, la résilience et le fonctionnement ont été évalués au début de l’étude et après 12 semaines.

**Résultats:**

L’acceptabilité du programme OHP pour les jeunes présentant un risque clinique élevé de psychose a été démontrée par un taux de présence élevé aux séances (n = 26; 86,7% ont assisté aux 9 séances) et par les scores de satisfaction des participants (CSQ-8 : moyenne = 28,35; écart-type = 3,68). La faisabilité de l’essai clinique de confirmation a été appuyée par un recrutement élevé sur 12 mois (n = 30; taux de 2 à 3 par mois), une rétention élevée (n = 26; 86,7% à 12 semaines), la fidélité à l’intervention et la facilité à respecter le calendrier d’évaluations cliniques. Les indicateurs préliminaires de l’efficacité clinique ont été démontrés par une diminution des symptômes psychiatriques, une amélioration de la résilience et un fonctionnement amélioré.

**Conclusions:**

Le programme OHP pour les jeunes présentant un risque clinique élevé de psychose est une intervention psychosociale prometteuse conçue conjointement avec des jeunes présentant un risque clinique élevé de psychose. Nos données appuient l’acceptabilité de l’intervention, la faisabilité de l’essai clinique de confirmation et les résultats préliminaires concernant l’efficacité sur le plan de l’amélioration des résultats cliniques et fonctionnels.

L’essai est enregistré sur le site ClinicalTrials.gov NCT05757128.

## Introduction

The clinical high risk for psychosis (CHR) construct is used to identify individuals at elevated risk of developing a primary psychotic disorder.^1–3^ Although CHR patients present with subthreshold psychotic symptoms that do not meet diagnostic criteria for a psychotic disorder, they still experience psychological distress, functional impairment, and reduced quality of life.^4^ Approximately 20% of CHR patients transition to a psychotic disorder within 3 years,^5,6^ but many others continue to experience persistent subthreshold psychotic symptoms, comorbid mood and anxiety disorders, and functional challenges.^4,7–9^

The clinical heterogeneity and varied trajectories within the CHR group have led to challenges in developing effective and scalable care models.^10–12^ Clinical trials in CHR, which are essential to inform the development of patient-centred, evidence-based services, have primarily focused on symptom reduction and the prevention of psychosis onset.^13^ However, more recent paradigm shifts have emphasized multicomponent, developmentally appropriate, and patient-centred approaches that prioritize broader psychosocial outcomes.^14,15^ In this context, transdiagnostic interventions that promote resilience, psychological well-being, and personal agency may serve as effective foundational strategies for this population, potentially laying the groundwork for subsequent, more intensive interventions tailored to individual clinical profiles.^15^

The Optimal Health Program (OHP) is a transdiagnostic, structured psychosocial intervention, which was originally developed for adults with severe mental illness. OHP is grounded in self-efficacy theory and designed to promote resilience, coping, and functional recovery through self-management.^16–19^ OHP has been shown to improve depression, anxiety, and quality of life in adult community dwelling mental health patients and in patients with chronic medical conditions.^16,20,21^ We engaged youth with lived experience and adapted OHP to the unique needs of CHR patients.^22^ OHP-CHR is a co-designed, developmentally appropriate intervention with the potential to improve clinical and functional outcomes for CHR youth. OHP-CHR retains the core structure and theoretical framework of the original programme while incorporating youth-friendly and recovery-oriented language, supportive activities, and a more engaging design. Informed by the stress-vulnerability model, OHP-CHR sessions focus on psychoeducation, coping strategies, relapse prevention, and health promotion.

Herein, we present the quantitative results of a study evaluating the acceptability of OHP-CHR, the feasibility of conducting a confirmatory clinical trial, and the preliminary efficacy in improving clinical and function outcomes in CHR youth.

We hypothesized that:

1. OHP-CHR would be acceptable as demonstrated by high attendance rates and high satisfaction scores on Client Satisfaction Questionnaire (CSQ-8).
2. A confirmatory clinical trial of OHP-CHR would be feasible as demonstrated by our ability to recruit our desired sample of 30 participants over 12 months, retain >80% at 12 weeks, have strong intervention adherence (>50% of participants attending at least 50% of sessions), high intervention fidelity, and no challenges in completing the assessment schedule.

## Methods

We report this study in alignment with the CONSORT 2010 extension for pilot and feasibility studies^23^ (see Supplemental Table S1 for checklist).

### Study Design

Our methods are described in detail in a published study protocol.^24^ In brief, this study was a single-arm clinical trial evaluating OHP-CHR using a pre–post-design. The trial was conducted at the Centre for Addiction and Mental Health (CAMH) in Toronto, Canada, and received ethics approval from the CAMH Research Ethics Board (REB number: 063/2022). The trial was registered with ClinicalTrials.gov (registration number: NCT05757128).

### Participants

*Participant recruitment.* Participants were recruited from the CHR Clinical Research programme at CAMH's Slaight Family Centre for Youth in Transition (SFCYT; Early psychosis intervention programme). Approximately two–three new patients are assessed per week by the CHR service and over a 100 followed in the programme at any point in time. All patients are approached for research participation at their first consultation and throughout their care. There primary mechanism of recruitment within the programme is through the Slaight centralized clinical research recruitment strategy, where a dedicated team of clinical research staff identify and engage patients receiving early psychosis services, facilitating streamlined triaging into appropriate studies. Patients who expressed interest in the study first underwent virtual screening meetings. After screening, potential participants attended an in-person evaluation for consent and comprehensive eligibility assessment. Participants received $30 in compensation for baseline assessments and $30 in compensation for end of study assessments, as approved by the CAMH Research Ethics Board. No compensation was provided to participants for attending the OHP-CHR intervention sessions.

*Inclusion and exclusion criteria.* Participants were eligible for the trial if they were between 16 and 29 years of age, currently met or had met within the past 3 years the criteria for at least one psychosis-risk syndrome as assessed by the Structured Interview for Psychosis-Risk Syndromes (SIPS),^25^ and had the capacity and willingness to provide informed consent. Participants were excluded if they had a Diagnostic and Statistical Manual of Mental Disorders, Fifth Edition (DSM-5) diagnosis of a psychotic disorder, a documented history of intellectual disability, or presented with acute suicidality requiring immediate intervention. All participants provided written informed consent to participate in the study.

### Study Intervention

OHP-CHR consists of three core components: (1) assessment and engagement, aimed at identifying potential barriers to treatment and establishing strategies to enhance participant engagement; (2) therapy sessions, grounded in stress-vulnerability and self-efficacy models, focusing on psychoeducation, coping strategies, relapse prevention, and skills to promote well-being, resilience, and sustaining mental health; and (3) maintenance integration, facilitated through an individualized journal, that enables participants to track stressors, early warning signs, coping strategies, support systems, and other factors influencing their mental health. The intervention is delivered over 12 weeks, comprising nine individual sessions (either in-person or virtual, based on participant preference), each lasting approximately 1 h. Sessions are held weekly for the first 6 weeks and every 2 weeks for the remaining 6 weeks. Participants are provided with a structured workbook to support ongoing skill-building. The facilitator delivering the programme completed a two-day training workshop and received supervision from Dr David Castle. A detailed description of OHP-CHR has been reported in our published study protocol^24^ and in the manuscript describing the process of adaptation through engagement with youth with lived experience.^22^

### Study Outcomes Measures

Baseline data included sociodemographic information, including age, gender, ethnoracial background, relationship status, employment and educational status, and living arrangements. Comorbid psychiatric disorders were assessed using the Structured Clinical Interview for DSM-5 (SCID-5),^26^ and the use of psychotropic medication at baseline was also recorded.

The primary outcomes were evaluating the acceptability of OHP-CHR and the feasibility of conducting a larger confirmatory clinical trial. Acceptability was informed by OHP session attendance rates and satisfaction scores using the CSQ-8.^27^ Based on standard thresholds, scores above 24 were considered indicative of high satisfaction.^27^ Feasibility was informed by recruitment, retention, adherence, and attrition rates. Recruitment was evaluated by calculating the number of participants who agreed to screening, met eligibility criteria, and were subsequently enrolled during the 12-month study period. Retention was measured as the proportion of participants who completed the 12-week trial. Attrition was calculated as the proportion of participants who withdrew prior to completing the 12-week study. Intervention fidelity was assessed using a session adherence checklist developed by our team that aligned with the core components of each session, and consistent with fidelity measures used in prior OHP trials.^21^ Each item was rated as either “completed” or “not completed” (Supplemental Table S2). Intervention fidelity of 80% or higher is considered high.^28^

Exploratory evaluation of clinical efficacy was informed by measures of psychiatric symptoms, resilience, and functioning, collected at baseline and at 12 weeks (end of intervention). Psychosis-risk symptoms were evaluated using the Scale of Psychosis-Risk Symptoms (SOPS)^25^ and PRIME-Revised^29^ with higher scores being indicative of higher symptom severity. Depressive symptoms were assessed using the Calgary Depression Scale for Schizophrenia (CDSS),^30^ a 9-item clinician-rated scale with 4-point Likert responses, where higher scores indicate greater severity. Anxiety symptoms were assessed with the State-Trait Anxiety Inventory (STAI),^31^ a 40-item self-report measure using a 4-point Likert scale, with higher scores reflecting greater anxiety. Resilience was assessed using the 25-item self-report Connor–Davidson Resilience Scale (CD-RISC),^32^ rated on a 5-point scale with total scores ranging from 0 to 100 and higher scores reflecting greater resilience. Functioning and disability were evaluated using the self-report WHO Disability Assessment Schedule 2.0 (WHO-DAS 2.0)^33^ and the Global Functioning (GF): Social and Role scales.^34^ WHO-DAS is a 12-item self-report measure using a 5-point Likert scale, with higher scores indicating greater disability across multiple domains of functioning. The GF scales are brief clinician-rated interviews with detailed anchors; scores range from 1 to 10, with higher scores indicating better functioning. Adverse events and serious adverse events were assessed by active monitoring at each study visit where participants were specifically asked about changes in health, hospitalizations, or safety concerns.

### Statistical Analyses

Baseline characteristics and follow-up outcomes were summarized using means and standard deviations for continuous variables, and frequencies with percentages for categorical variables. To evaluate the potential impact of missing data, baseline characteristics were compared between participants who completed all scheduled assessments and those who dropped out. Group comparisons at baseline were conducted using Fisher's exact test for binary variables, and Student's *t*-test for parametric continuous variables. Feasibility outcomes were assessed as count and proportion data. Intervention fidelity was calculated based on adherence to each checklist item across all participants. For each component, adherence was defined as the proportion of participants for whom the component was delivered. An overall fidelity score was then derived by averaging adherence rates across all checklist items.

Changes in exploratory clinical outcomes (psychosis-risk symptoms, depression, anxiety, functioning, and resilience) pre- to post-intervention were described using generalized linear mixed-effects models, which account for missing data and within-participant correlations over time. Baseline score for the variable of interest and time were included as the fixed effects with random participant effect included in the model. Mixed-effects model analyses were also performed controlling for age, gender, attendance method, and baseline antipsychotic treatment. Given the preliminary nature of this study, we calculated changes in exploratory outcomes for descriptive purposes, rather than for hypothesis testing of clinical effects. All statistical analyses were conducted using IBM Statistical Package for the Social Sciences (SPSS) version 27.

## Results

### Study Participants

Baseline characteristics are described in Table 1. The mean age of the sample was 21.9 years. The sample was 50% female, and most participants identified as White (46.7%). Most participants (86.7%; *n* = 26) met criteria for attenuated positive-symptom risk syndrome (APSS), three met criteria for both APSS and genetic risk and deterioration syndrome, and one met criterion for brief intermittent psychotic syndrome. Concurrent SCID diagnoses included major depressive disorder (40%; *n* = 12), anxiety disorders (26.7%; *n* = 8), substance use disorders (26.7%; *n* = 8), obsessive-compulsive disorder (16.7%; *n* = 5), eating disorders (10%; *n* = 3), and post-traumatic stress disorder (6.7%; *n* = 2). A first-degree family history of psychosis was reported by 16.7% (*n* = 5). At baseline, 43.3% (*n* = 13) of participants were receiving antidepressants alone, 3.3% (*n* = 1) were taking antipsychotic medication alone, 30% (*n* = 9) were taking both antidepressants and antipsychotics, and 23.3% (*n* = 7) were not taking either antidepressant or antipsychotics. None of the participants were receiving any concurrent psychosocial interventions during study participation. No significant differences were observed between participants who completed the study (*n* = 26) and those who dropped out (*n* = 4) in terms of sociodemographic factors, SOPS positive and negative symptoms, or social and role functioning (Supplemental Table S3).

**Table 1.** Baseline Demographic and Clinical Characteristics of Study Participants (*n* = 30)^a^.

Variables	*n* (%)
Age in years (mean (SD))	21.9 (3.7)
Gender identity	
Man	10 (33.3%)
Woman	15 (50.0%)
Non-binary or prefer not to answer	5 (16.7%)
Ethnoracial background	
White	14 (46.7%)
East Asian	7 (23.3%)
South Asian	4 (13.3%)
Others	5 (16.7%)
Relationship status	
Single	21 (70.0%)
In a relationship	9 (30.0%)
Living arrangement	
Living with family/partner	24 (80.0%)
Living on own/with roommate	6 (20.0%)
Current employment/education	
Full-time/part-time student	9 (30.0%)
Full-time/part-time employment	9 (30.0%)
Combination of school and work	3 (10.0%)
Not working/not in school	9 (30.0%)
Concurrent SCID diagnosis	
Diagnosis	
Major depressive disorder	12 (40%)
Anxiety disorder	8 (26.7%)
Eating disorder	3 (10.0%)
Obsessive compulsive disorder	5 (16.7%)
Substance use disorder	8 (26.7%)
Post-traumatic stress disorder	2 (6.7%)
Number of diagnoses	
Single diagnosis	9 (30%)
Multiple diagnoses	13 (43.3%)
No diagnosis	8 (26.7%)
First degree family history of psychosis	5 (16.7%)
Medication	
Antidepressant treatment alone	13 (43.3%)
Antipsychotic treatment alone	1 (3.3%)
Both antidepressant and antipsychotic treatment	9 (30%)
No medication	7 (23.3%)

*Note*. SCID = Structured Clinical Interview for DSM.

a Characteristics are shown as *n* (%) unless otherwise specified.

### Feasibility and Acceptability

**Figure 1.**
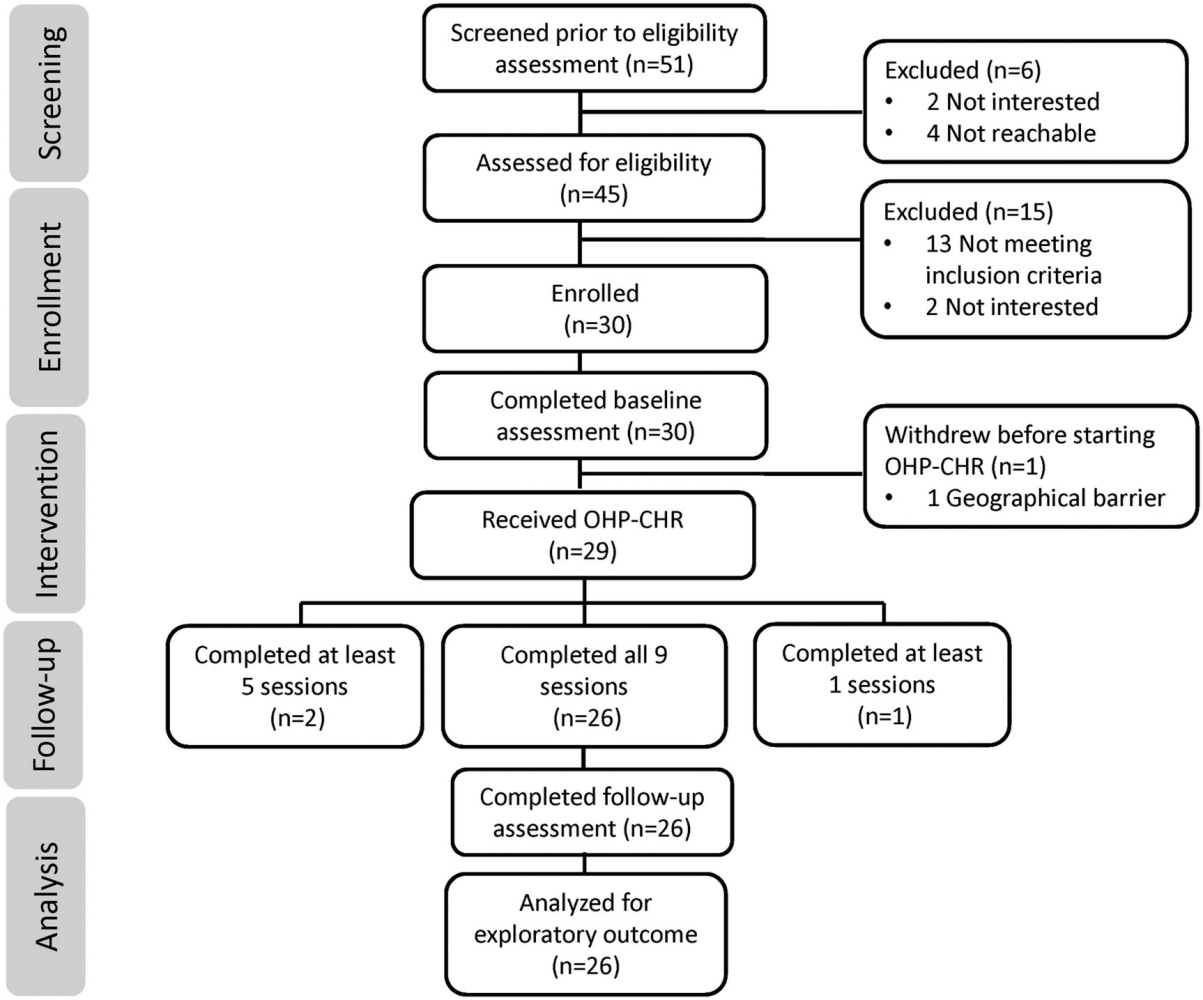
Consort diagram.

Between September 2023 and August 2024, 51 patients were referred to the study, 45 (88.2%) expressed interest and were contacted by research staff for eligibility screening. Two individuals declined participation after initial expression of interest, and 13 did not meet inclusion criteria. The eligibility rate was 70.0% with 30 of 43 individuals meeting inclusion criteria and the remaining 13 were excluded for not meeting CHR criteria. All 30 eligible participants were enrolled in the study over a 12-month period, resulting in a recruitment rate of approximately 2.5 participants per month. Of the 30 enrolled, 29 initiated the intervention, and one dropped out prior to starting. Retention within the trial was very good with 26 participants (86.7%) completing the study. Three withdrew after initiating the OHP intervention, yielding an attrition rate of 13.3% (4 of 30). Of the 30 enrolled participants, 28 (93.3%) completed at least five sessions, and 26 (86.7%) completed all nine sessions along with post-intervention assessments. Among those who began the intervention, 51.7% (15 of 29) attended sessions virtually, 41.4% (12 of 29) attended in person, and 6.9% (2 of 29) participated in a hybrid format. No safety concerns or serious adverse events were reported. Recruitment and follow-up details are illustrated in the CONSORT diagram (Figure 1). Mean fidelity to OHP-CHR was 91.8% (SD 10.5%). Details on fidelity for each component can be found in Supplemental Table S2. All 30 enrolled participants completed baseline assessments, with 93.3% (*n* = 28) doing so in one visit. At baseline, 86.7% (*n* = 26) had complete data. Missing data included four cases missing PRIME-R data, and two cases each of missing WHO-DAS, STAI-S, STAI-T, and CD-RISC 25 data. At 12 weeks, 26 completed follow-up. Of these, 96.2% (25 of 26) had complete data. Missing outcome data included one case missing WHO-DAS data. Missing outcome data represented 4.3% of data points at baseline and 0.4% at 12 weeks.

**Figure 2.**
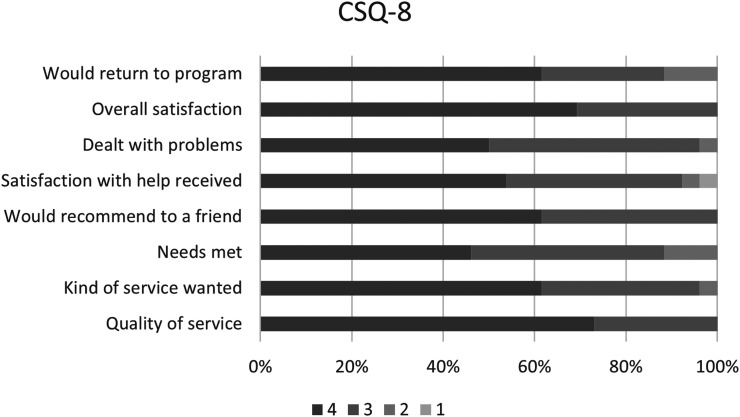
Participant satisfaction as measured by the Client Satisfaction Questionnaire (CSQ-8).

The CSQ-8 was completed by 26 participants, with total scores ranging from 20 to 32 (*M* = 28.35, SD = 3.68) (Table 2). High satisfaction was reported by 24 out of 26 participants (92.3%). All respondents rated the quality of services as either good or excellent, reported being very or mostly satisfied, and indicated they would recommend the programme to a friend. Additionally, 96% (25 of 26) agreed that they received the kind of services they wanted and felt the programme helped them address their problems more effectively. Overall, 92% (24 of 26) reported being very or mostly satisfied, and 88% (*n* = 23) indicated that most or almost all their needs were met and that they would return for help if needed (Figure 2).

**Table 2.** Participant Ratings on the Client Satisfaction Questionnaire (CSQ-8) on a Scale of 1–5.

CSQ-8	Participants (*n* = 26), Mean (SD)
1. Quality of service	3.73 (0.45)
2. Kind of service wanted	3.58 (0.58)
3. Needs met	3.34 (0.69)
4. Would recommend to friend	3.61 (0.50)
5. Satisfaction with help received	3.42 (0.76)
6. Dealt with problems	3.46 (0.58)
7. Overall satisfaction	3.69 (0.47)
8. Would return to programme	3.50 (0.71)
Total score (range 8–32)	28.35 (3.68)

### Exploratory Clinical and Functional Outcomes

Exploratory linear mixed-effects modelling was conducted on clinical outcomes for the 26 participants who completed both pre- and post-intervention assessments. On the SIPS, positive symptoms significantly decreased by 2.88 points (95% confidence interval (CI), −4.39 to −1.38), and negative symptoms decreased by 1.61 points (95% CI, −2.82 to −0.42). No significant changes were observed in disorganized symptoms (β = −0.31, 95% CI, −0.92 to 0.30) or general symptoms (β = −0.77, 95% CI, −1.71 to 0.17). Depressive symptoms, as measured by the CDSS, decreased by 1.96 points (95% CI, −3.50 to −0.46). Anxiety symptoms also improved, with State-Trait Anxiety Inventory-State scale (STAI-S) scores decreasing by 8.92 points (95% CI, −12.21 to −5.63) and State-Trait Anxiety Inventory-Trait scale (STAI-T) scores decreasing by 7.48 points (95% CI, −10.70 to −4.25). Resilience, assessed by CD-RISC 25, increased by 10.30 points (95% CI, 5.14 to 15.47) following the intervention. In terms of functioning, role functioning improved by 0.88 points on GF:R (95% CI, 0.46 to 1.31), and disability measured by the WHO-DAS decreased by 4.40 points (95% CI, −6.83 to −1.97). Social functioning as measured by GF:S did not show a significant change (β = 0.35, 95% CI, 0.00 to 0.72). These results can be found in Table 3. Mixed model analyses were repeated while controlling for age, gender, attendance methods, and baseline antipsychotic treatment. The results remained consistent.

**Table 3.** Differences in Variables Within Group Pre- and Post-intervention (*n* = 26).

Variable	Baseline		End of Treatment				
	*M*	SE	*M*	SE	Beta	95% CI	Hedges’ *g*
PRIME-R	38.59	3.08	28.35	3.39	−10.11	−2.45 to −17.77	1.055
SIPS-Pos	10.96	0.80	8.08	0.77	−2.88	−1.38 to −4.39	0.746
SIPS-Neg	10.92	0.97	9.31	1.14	−1.61	−0.42 to −2.82	0.522
SIPS-Dis	5.46	0.64	5.15	0.67	−0.31	−0.92 to 0.30	0.196
SIPS-Gen	9.35	0.63	8.58	0.78	−0.77	−1.71 to 0.17	0.317
CDSS	5.58	0.81	3.62	0.70	−1.96	−3.50 to −0.46	0.995
STAI-S	55.08	2.31	46.15	2.35	−8.92	−5.63 to −12.21	1.050
STAI-T	61.05	2.02	53.58	2.38	−7.48	−10.70 to −4.25	0.890
WHO-DAS	18.92	1.71	14.52	1.64	−4.40	−6.83 to −1.97	1.150
GF:R	5.73	0.34	6.62	0.38	0.88	0.46 to 1.31	−0.810
GF:S	6.45	0.27	6.80	0.23	0.35	0.00 to 0.72	−0.378
CD-RISC 25	45.74	3.14	56.04	4.05	10.30	5.14 to 15.47	−0.758

*Note*. SIPS = Structured Interview for Psychosis-Risk Syndromes; CDSS = Calgary Depression Scale for Schizophrenia; STAI-S = State-Trait Anxiety Inventory-State scale; STAI-T = State-Trait Anxiety Inventory-Trait scale; WHO-DAS = WHO Disability Assessment Schedule; GF:R = Global Functioning-Role; GF:S = Global Functioning-Social; CD-RISC = Connor–Davidson Resilience Scale.

There were no participants who transitioned to psychosis by the end of the study based on the SIPS at 12 weeks.

## Discussion

This study evaluated OHP-CHR, a novel psychosocial intervention, specifically designed to meet the needs and priorities of CHR youth. Our data support that OHP-CHR is acceptable to CHR youth as indicated by high session attendance rates and participant satisfaction scores. A confirmatory clinical trial is feasible given the robust recruitment, achieving our target sample of 30 participants within 12 months and retaining over 80% at study completion. Additional data supporting feasibility are high session attendance rates, with 86.7% completing all nine sessions, and no challenges being encountered in completing the assessment schedule. Intervention fidelity measures were also favourable additionally supporting feasibility of a larger clinical trial.

Retention rates in the present trial exceed what is typically observed in CHR trials where approximately one-third participants drop out prior to study completion.^35^ Attendance rates were also greater than many previously reported psychosocial interventions trials in CHR populations.^36–39^ Strong retention and attendance rates ensure participants receive the intended treatment and strengthen the interpretability of outcomes.^40,41^ The co-development of OHP-CHR with lived experience partners from the Youth Engagement Initiative at CAMH likely played a central role in enhancing acceptability to CHR youth.^22^ Youth with lived experience provided meaningful input into the content and design of OHP-CHR, helping ensure that the programme was developmentally appropriate, engaging, and aligned with the needs and preferences of the intended audience. A growing body of literature indicates that integration of lived experience in psychosocial intervention development enhances engagement and contextual fit.^42,43^ OHP-CHR takes a patient-centred and strength-based approach, emphasizing well-being rather than focusing solely on symptom reduction, which may align more with CHR youth priorities.^44^ The flexible delivery format of our intervention, offered in person or virtually, potentially also reduced logistical barriers and supported participant engagement. Additional insights into the factors contributing to the acceptability of OHP-CHR will be informed by end of study qualitative interviews examining participants’ overall perception of the intervention, and the barriers/facilitators to engagement.

We have been able to demonstrate that we can recruit individuals from under-represented groups, including ethnic minority and gender diverse participants. Although ethnic minority groups are under-represented in clinical research, approximately half of our sample were from diverse ethnoracial backgrounds, broadly reflective of the demographic composition of urban centres in Canada. Minority representation was supported by the integrated clinical research setting, centralized recruitment infrastructure, and institutional-level equity, diversity, and inclusion (EDI) initiatives at our centre that ensure equitable access to research participation. These system-level approaches align with frameworks that prioritize EDI considerations across the lifecycle of clinical research and enhance participation of under-represented groups.^45,46^ Consistent with most CHR clinical research studies, the majority of participants met criteria for APSS and many had concurrent psychiatric diagnoses including major depressive disorder, anxiety disorders, substance use disorders, obsessive-compulsive disorder, eating disorders, and post-traumatic stress disorder, reflecting the high rates of mental health comorbidity commonly observed in CHR youth.^47^ The characteristics of our sample support our ability to recruit a representative sample to CHR clinical research studies.

Participants showed improvements in attenuated psychotic symptoms, negative symptoms, depression and anxiety, role functioning, disability, and resilience after engaging in OHP-CHR. While gains were noted on the WHO-DAS and GF: Role, no improvement was observed on the GF: Social. A very recent meta-analysis evaluated the efficacy of interventions in reducing risk of transition to psychosis, attenuated psychotic symptoms, and overall functioning in CHR and found no evidence of efficacy of any intervention when compared to control conditions.^11^ The authors argued that novel treatment approaches need to be evaluated in CHR.^11^ Few CHR trials have directly examined resilience, which may represent a meaningful intervention target.^48^ Resilience may serve as an important outcome for interventions aiming to enhance protective factors and avert negative trajectories in this population.^49^ In the present study, participants showed improved resilience and functioning, indicating that OHP-CHR may have potential as a protective psychosocial intervention in CHR.^49,50^

This study was not designed to formally test hypotheses related to clinical effect, and without a comparator group it is difficult to conclude that the observed improvements in clinical outcomes are secondary to the OHP intervention or related to the natural course of CHR illness trajectory. It is worth noting that although approximately 30% of CHR individuals will remit spontaneously, the majority continue to experience persistent and distressing psychosis spectrum symptoms, in addition to ongoing functional impairment.^8,9^ High attendance and satisfaction rates are indicators that an intervention is acceptable, implementable, and likely to yield meaningful clinical outcomes.^14,51^ Unsurprisingly, consistent participation in psychosocial treatment predicts better clinical outcomes.^52–54^ We would argue that the potential therapeutic benefits of OHP-CHR do warrant further evaluation in future rigorously designed clinical trials.

The findings of this study need to be considered in the context of several limitations. Participants agreeing to the study may represent only a subset of CHR youth who are more motivated to engage in clinical research, potentially inflating acceptability indices for OHP-CHR. The bias introduced by unblinded assessors and participants must be acknowledged. Therapeutic alliance may have influenced participant engagement and outcomes. Gold standard approaches^55^ to assessing fidelity were not employed in the present study. Future trials should incorporate independent evaluation of recorded sessions to ensure adherence to the components of OHP and intervention fidelity. The primary focus of the study was to evaluate acceptability of the OHP-CHR and the feasibility of conducting a confirmatory clinical trial, therefore, our ability to draw causal conclusions about clinical efficacy is limited. Although not formally assessed, we identified that youth-orientated, recovery focused content in a flexibly delivered intervention may support implementation by facilitating engagement and promoting adherence.

In conclusion, the findings of the current study support that OHP-CHR is an acceptable intervention to CHR youth and has promise as a developmentally appropriate, youth-friendly intervention. Future confirmatory studies are feasible and warranted to rigorously evaluate the clinical efficacy of OHP-CHR along with pathways to larger scale implementation and adoption.

## Supplemental Material

sj-doc-1-cpa-10.1177_07067437261436536 - Supplemental material for Optimizing Health for Canadian Youth at Clinical High Risk for Psychosis: A Feasibility Study: Optimiser la santé des jeunes Canadiens présentant un risque clinique élevé de psychose : Une étude de faisabilité Supplemental material, sj-doc-1-cpa-10.1177_07067437261436536 for Optimizing Health for Canadian Youth at Clinical High Risk for Psychosis: A Feasibility Study: Optimiser la santé des jeunes Canadiens présentant un risque clinique élevé de psychose : Une étude de faisabilité by Yun Lu, Beatrice Todesco, Lisa D. Hawke, Nicole Kozloff, Gillian Strudwick, Michael Kiang, Wei Wang, David Castle and George Foussias, Muhammad Omair Husain in The Canadian Journal of Psychiatry

## References

[1] Fusar-Poli P Salazar de Pablo G Correll CU , et al. Prevention of psychosis: advances in detection, prognosis, and intervention. JAMA Psychiatry. 2020;77(7):755–765. doi:10.1001/jamapsychiatry.2019.4779 32159746

[2] Addington J Addington D Abidi S Raedler T Remington G . Canadian treatment guidelines for individuals at clinical high risk of psychosis. Can J Psychiatry. 2017;62(9):656–661. doi:10.1177/0706743717719895 28730848 PMC5593244

[3] Yung AR Yuen HP McGorry PD , et al. Mapping the onset of psychosis: the comprehensive assessment of at-risk mental states. Aust N Z J Psychiatry. 2005;39(11–12):964–971. doi:10.1080/j.1440-1614.2005.01714.x 16343296

[4] Allswede DM Addington J Bearden CE , et al. Characterizing covariant trajectories of individuals at clinical high risk for psychosis across symptomatic and functional domains. Am J Psychiatry. 2020;177(2):164–171. doi:10.1176/appi.ajp.2019.18111290 31509005 PMC7002249

[5] Nelson B Yuen HP Wood SJ , et al. Long-term follow-up of a group at ultra high risk (“prodromal”) for psychosis: the PACE 400 study. JAMA Psychiatry. 2013;70(8):793–802. doi:10.1001/jamapsychiatry.2013.1270 23739772

[6] Salazar de Pablo G Radua J Pereira J , et al. Probability of transition to psychosis in individuals at clinical high risk: an updated meta-analysis. JAMA Psychiatry. 2021;78(9):970–978. doi:10.1001/jamapsychiatry.2021.0830 34259821 PMC8281006

[7] Lin A Wood SJ Nelson B Beavan A McGorry P Yung AR . Outcomes of nontransitioned cases in a sample at ultra-high risk for psychosis. Am J Psychiatry. 2015;172(3):249–258. doi:10.1176/appi.ajp.2014.13030418 25727537

[8] Beck K Andreou C Studerus E , et al. Clinical and functional long-term outcome of patients at clinical high risk (CHR) for psychosis without transition to psychosis: a systematic review. Schizophr Res. 2019;210:39–47. doi:10.1016/j.schres.2018.12.047 30651204

[9] Simon AE Borgwardt S Riecher-Rössler A Velthorst E de Haan L Fusar-Poli P . Moving beyond transition outcomes: meta-analysis of remission rates in individuals at high clinical risk for psychosis. Psychiatry Res. 2013;209(3):266–272. doi:10.1016/j.psychres.2013.03.004 23871169

[10] Davies C Radua J Cipriani A , et al. Efficacy and acceptability of interventions for attenuated positive psychotic symptoms in individuals at clinical high risk of psychosis: a network meta-analysis. Front Psychiatry. 2018;9:187. doi:10.3389/fpsyt.2018.00187 29946270 PMC6005890

[11] Minichino A Davies C Karpenko O , et al. Preventing psychosis in people at clinical high risk: an updated meta-analysis by the World Psychiatric Association Preventive Psychiatry section. Mol Psychiatry. 2025;30(6):2773–2782. doi:10.1038/s41380-025-02902-8 39953286 PMC12092282

[12] Bosnjak Kuharic D Kekin I Hew J Rojnic Kuzman M Puljak L . Interventions for prodromal stage of psychosis. Cochrane Database Syst Rev. 2019;2019(11):CD012236. doi:10.1002/14651858.CD012236.pub2 PMC6823626 31689359

[13] Mei C van der Gaag M Nelson B , et al. Preventive interventions for individuals at ultra high risk for psychosis: an updated and extended meta-analysis. Clin Psychol Rev. 2021;86:102005. doi:10.1016/j.cpr.2021.102005 33810885

[14] Castle D Hawke L Henderson J Husain MO Lusicic A Szatmari P . Complex interventions for youth mental health: a way forward. Can J Psychiatry. 2022;67(10):755–757. doi:10.1177/07067437221093396 35484783 PMC9510997

[15] Hamilton SA Wastler HM Moe AM , et al. Symptomatic and functional outcomes among individuals at high risk for psychosis participating in step-based care. Psychiatr Serv. 2024;75(5):496–499. doi:10.1176/appi.ps.20230188 38088038

[16] Gilbert MM Chamberlain JA White CR , et al. Controlled clinical trial of a self-management program for people with mental illness in an adult mental health service—the Optimal Health Program (OHP). Aust Health Rev. 2012;36(1):1–7. doi:10.1071/AH11008 22513012

[17] Ferrier L Ski CF O'Brien C , et al. Bridging the gap between diabetes care and mental health: perspectives of the Mental health IN DiabeteS Optimal Health Program (MINDS OHP). BMC Endocr Disord. 2021;21(1):96. doi:10.1186/s12902-021-00760-3 33964904 PMC8105945

[18] Minshall C Castle DJ Thompson DR , et al. A psychosocial intervention for stroke survivors and carers: 12-month outcomes of a randomized controlled trial. Top Stroke Rehabil. 2020;27(8):563–576. doi:10.1080/10749357.2020.1738677 32191569

[19] Jenkins ZM Tan EJ O'Flaherty E , et al. A psychosocial intervention for individuals with advanced chronic kidney disease: a feasibility randomized controlled trial. Nephrology (Carlton). 2021;26(5):442–453. doi:10.1111/nep.13850 33484221

[20] Sheth MS Castle DJ Wang W Lee A Jenkins ZM Hawke LD . Changes to coping and its relationship to improved wellbeing in the optimal health program for chronic disease. SSM—Mental Health. 2023;3:100190. doi:10.1016/j.ssmmh.2023.100190

[21] O’Brien CL Apputhurai P Knowles SR , et al. Initial evaluation of the Optimal Health Program for people with diabetes: 12-month outcomes of a randomised controlled trial. Psychol Health. 2024;39(3):358–378. doi:10.1080/08870446.2022.2060507 35465777

[22] Lu Y Hedemann TL Hawke LD , et al. Adaptation of a psychosocial intervention for Canadian youth at clinical high risk for psychosis [Adaptation d'une intervention psychosociale pour les jeunes à haut risque clinique de psychose au Canada]. Can J Psychiatry. 2025;70(12):887–895. doi:10.1177/07067437251328357 40221981 PMC11994639

[23] Eldridge SM Chan CL Campbell MJ , et al. CONSORT 2010 statement: extension to randomised pilot and feasibility trials. Pilot Feasibility Stud. 2016;2:64. doi:10.1186/s40814-016-0105-8 27965879 PMC5154046

[24] Husain MO Hawke LD Lu Y , et al. A mixed-methods study to evaluate the feasibility and preliminary efficacy of delivering the optimal health program (OHP) for youth at clinical high risk (CHR) for psychosis: a study protocol. PLoS One. 2024;19(7):e0306968. doi:10.1371/journal.pone.0306968 PMC11257342 39024237

[25] Miller TJ McGlashan TH Rosen JL , et al. Prodromal assessment with the structured interview for prodromal syndromes and the scale of prodromal symptoms: predictive validity, interrater reliability, and training to reliability. Schizophr Bull. 2003;29(4):703–715. doi:10.1093/oxfordjournals.schbul.a007040 14989408

[26] First MB Spitzer RL Gibbon M Williams JB . Structured clinical interview for DSM-IV-TR axis I disorders, research version, patient edition. 2002.

[27] Attkisson CC Zwick R . The Client Satisfaction Questionnaire. Psychometric properties and correlations with service utilization and psychotherapy outcome. Eval Program Plann. 1982;5(3):233–237. doi:10.1016/0149-7189(82)90074-x 10259963

[28] Hill HC Erickson A . Using implementation fidelity to aid in interpreting program impacts: a brief review. Educ Res. 2019;48(9):590–598. doi:10.3102/0013189(19891436

[29] Miller TJ Zipursky RB Perkins D , et al. The PRIME North America randomized double-blind clinical trial of olanzapine versus placebo in patients at risk of being prodromally symptomatic for psychosis. II. Baseline characteristics of the “prodromal” sample. Schizophr Res. 2003;61(1):19–30. doi:10.1016/s0920-9964(02)00440-1 12648732

[30] Addington D Addington J Schissel B . A depression rating scale for schizophrenics. Schizophr Res. 1990;3(4):247–251. doi:10.1016/0920-9964(90)90005-r 2278986

[31] Cao Y Liu Z . Factor structure and factorial invariance of the State-Trait Anxiety Inventory for Chinese children and adolescents. Psych J. 2015;4(2):74–87. doi:10.1002/pchj.78 26261907

[32] Connor KM Davidson JR . Development of a new resilience scale: the Connor–Davidson Resilience Scale (CD-RISC). Depress Anxiety. 2003;18(2):76–82. doi:10.1002/da.10113 12964174

[33] Kimber M Rehm J Ferro MA . Measurement invariance of the WHODAS 2.0 in a population-based sample of youth. PLoS One. 2015;10(11):e0142385. doi:10.1371/journal.pone.0142385 PMC4643914 26565410

[34] Cornblatt BA Auther AM Niendam T , et al. Preliminary findings for two new measures of social and role functioning in the prodromal phase of schizophrenia. Schizophr Bull. 2007;33(3):688–702. doi:10.1093/schbul/sbm029 17440198 PMC2526147

[35] Farris MS Devoe DJ Addington J . Attrition rates in trials for adolescents and young adults at clinical high-risk for psychosis: a systematic review and meta-analysis. Early Interv Psychiatry. 2020;14(5):515–527. doi:10.1111/eip.12864 31422583 PMC7025923

[36] Morrison AP French P Stewart SL , et al. Early detection and intervention evaluation for people at risk of psychosis: multisite randomised controlled trial. Br Med J. 2012;344:e2233. doi:10.1136/bmj.e2233 PMC3320714 22491790

[37] van der Gaag M Nieman DH Rietdijk J , et al. Cognitive behavioral therapy for subjects at ultrahigh risk for developing psychosis: a randomized controlled clinical trial. Schizophr Bull. 2012;38(6):1180–1188. doi:10.1093/schbul/sbs105 22941746 PMC3494039

[38] Addington J Epstein I Liu L French P Boydell KM Zipursky RB . A randomized controlled trial of cognitive behavioral therapy for individuals at clinical high risk of psychosis. Schizophr Res. 2011;125(1):54–61. doi:10.1016/j.schres.2010.10.015 21074974

[39] McGorry PD Mei C Amminger GP , et al. A sequential adaptive intervention strategy targeting remission and functional recovery in young people at ultrahigh risk of psychosis: the staged treatment in early psychosis (STEP) sequential multiple assignment randomized trial. JAMA Psychiatry. 2023;80(9):875–885. doi:10.1001/jamapsychiatry.2023.1947 37378974 PMC10308298

[40] Becker KD Buckingham SL Rith-Najarian L Kline ER . The common elements of treatment engagement for clinically high-risk youth and youth with first-episode psychosis. Early Interv Psychiatry. 2016;10(6):455–467. doi:10.1111/eip.12283 26486257

[41] Thabane L Ma J Chu R , et al. A tutorial on pilot studies: the what, why and how. BMC Med Res Methodol. 2010;10:1. doi:10.1186/1471-2288-10-1 20053272 PMC2824145

[42] Hawke LD Sheikhan NY Bastidas-Bilbao H Rodak T . Experience-based co-design of mental health services and interventions: a scoping review. SSM—Mental Health. 2024;5:100309. 10.1016/j.ssmmh.2024.100309

[43] Veldmeijer L Terlouw G Van Os J Van Dijk O Van ‘t Veer J Boonstra N . The involvement of service users and people with lived experience in mental health care innovation through design: systematic review. JMIR Ment Health. 2023;10:e46590. doi:10.2196/46590 PMC10410372 37490326

[44] Petros N Cullen AE Vieira S , et al. Examining service-user perspectives for the development of a good outcome checklist for individuals at clinical high risk for psychosis. Early Interv Psychiatry. 2021;15(3):606–615. doi:10.1111/eip.12991 32453511

[45] Gedela K Wong R Balendra S , et al. Embedding equity, diversity and inclusion processes within clinical trials and health and social care research. BMJ Open. 2025;15(3):e091807. doi:10.1136/bmjopen-2024-091807 PMC11956285 40147995

[46] Mishra SR Tan AC Waller K Lindley RI Webster AC . Conceptualizing, operationalizing, and utilizing equity, diversity, and inclusion in clinical trials: a scoping review. J Clin Epidemiol. 2025;179:111649. doi:10.1016/j.jclinepi.2024.111649 39710302

[47] Solmi M Soardo L Kaur S , et al. Meta-analytic prevalence of comorbid mental disorders in individuals at clinical high risk of psychosis: the case for transdiagnostic assessment. Mol Psychiatry. 2023;28(6):2291–2300. doi:10.1038/s41380-023-02029-8 37296309 PMC10611568

[48] Marulanda S Addington J . Resilience in individuals at clinical high risk for psychosis. Early Interv Psychiatry. 2016;10(3):212–219. doi:10.1111/eip.12174 25234104 PMC5037439

[49] Cadenhead KS Addington J Bearden CE , et al. Protective factors predict resilient outcomes in clinical high-risk youth with the highest individualized psychosis risk scores. Schizophr Bull. 2024:sbae182. doi:10.1093/schbul/sbae182 PMC13645835 39488001

[50] Nesbitt AE Sabiston CM deJonge ML Barbic SP Kozloff N Nalder EJ . A scoping review of resilience among transition-age youth with serious mental illness: tensions, knowledge gaps, and future directions. BMC Psychiatry. 2023;23(1):660. doi:10.1186/s12888-023-05158-0 37679708 PMC10483804

[51] Kistin C Silverstein M . Pilot studies: a critical but potentially misused component of interventional research. JAMA. 2015;314(15):1561–1562. doi:10.1001/jama.2015.10962 26501530 PMC4917389

[52] DiMatteo MR Giordani PJ Lepper HS Croghan TW . Patient adherence and medical treatment outcomes: a meta-analysis. Med Care. 2002;40(9):794–811. doi:10.1097/00005650-200209000-00009 12218770

[53] McLeod BD . Relation of the alliance with outcomes in youth psychotherapy: a meta-analysis. Clin Psychol Rev. 2011;31(4):603–616. doi:10.1016/j.cpr.2011.02.001 21482319

[54] Flückiger C Del Re AC Wampold BE Horvath AO . The alliance in adult psychotherapy: a meta-analytic synthesis. Psychotherapy (Chic). 2018;55(4):316–340. doi:10.1037/pst0000172 29792475

[55] Rookes TA Schrag A Walters K Armstrong M . Measures of fidelity of delivery and engagement in self-management interventions: a systematic review of measures. Clin Trials. 2022;19(6):665–672. doi:10.1177/17407745221118555 36017707 PMC9679554

