# Supplementary material for: Optimizing Health for Canadian Youth at Clinical High Risk for Psychosis: A Feasibility Study: Optimiser la santé des jeunes Canadiens présentant un risque clinique élevé de psychose : Une étude de faisabilité
Source: Can J Psychiatry. 2026 Apr 7:07067437261436536. Online ahead of print. doi: 10.1177/07067437261436536 (PMC13056807; doi:10.1177/07067437261436536)
Supplement: sj-doc-1-cpa-10.1177_07067437261436536 - Supplemental material for Optimizing Health for Canadian Youth at Clinical High Risk for Psychosis: A Feasibility Study: Optimiser la santé des jeunes Canadiens présentant un risque clinique élevé de psychose : Une étude de faisabilité [file sj-doc-1-cpa-10.1177_07067437261436536.doc]

Supplementary Material

Table S1. CONSORT 2010 checklist of information to include when reporting a pilot or feasibility trial

| Section/Topic | Item No | Checklist item | Reported on page No |
| --- | --- | --- | --- |
| Title and abstract | | | |
|  | 1a | Identification as a pilot or feasibility randomised trial in the title | Page 1 |
|  | 1b | Structured summary of pilot trial design, methods, results, and conclusions (for specific guidance see CONSORT abstract extension for pilot trials) | Page 2 |
| Introduction | | | |
| Background and objectives | 2a | Scientific background and explanation of rationale for future definitive trial, and reasons for randomised pilot trial | Page 3-4 |
|  | 2b | Specific objectives or research questions for pilot trial | Page 4 |
| Methods | | | |
| Trial design | 3a | Description of pilot trial design (such as parallel, factorial) including allocation ratio | Page 5 |
|  | 3b | Important changes to methods after pilot trial commencement (such as eligibility criteria), with reasons | NA |
| Participants | 4a | Eligibility criteria for participants | Page 6 |
|  | 4b | Settings and locations where the data were collected | Page 6 |
|  | 4c | How participants were identified and consented | Page 6 |
| Interventions | 5 | The interventions for each group with sufficient details to allow replication, including how and when they were actually administered | Page 6,7 |
| Outcomes | 6a | Completely defined prespecified assessments or measurements to address each pilot trial objective specified in 2b, including how and when they were assessed | Page 7 |
|  | 6b | Any changes to pilot trial assessments or measurements after the pilot trial commenced, with reasons | NA |
|  | 6c | If applicable, prespecified criteria used to judge whether, or how, to proceed with future definitive trial | Page 4 |
| Sample size | 7a | Rationale for numbers in the pilot trial | Page 5 |
|  | 7b | When applicable, explanation of any interim analyses and stopping guidelines | NA |
| Randomisation: |  |  |  |
| Sequence  generation | 8a | Method used to generate the random allocation sequence | NA |
|  | 8b | Type of randomisation(s); details of any restriction (such as blocking and block size) | NA |
| Allocation  concealment  mechanism | 9 | Mechanism used to implement the random allocation sequence (such as sequentially numbered containers), describing any steps taken to conceal the sequence until interventions were assigned | NA |
| Implementation | 10 | Who generated the random allocation sequence, who enrolled participants, and who assigned participants to interventions | NA |
| Blinding | 11a | If done, who was blinded after assignment to interventions (for example, participants, care providers, those assessing outcomes) and how | NA |
|  | 11b | If relevant, description of the similarity of interventions | NA |
| Statistical methods | 12 | Methods used to address each pilot trial objective whether qualitative or quantitative | Page 8,9 |
| Results | | | |
| Participant flow (a diagram is strongly recommended) | 13a | For each group, the numbers of participants who were approached and/or assessed for eligibility, randomly assigned, received intended treatment, and were assessed for each objective | Figure 1, Page 10 |
|  | 13b | For each group, losses and exclusions after randomisation, together with reasons | Figure 1 |
| Recruitment | 14a | Dates defining the periods of recruitment and follow-up | Page 10 |
|  | 14b | Why the pilot trial ended or was stopped | NA |
| Baseline data | 15 | A table showing baseline demographic and clinical characteristics for each group | Table 1 |
| Numbers analysed | 16 | For each objective, number of participants (denominator) included in each analysis. If relevant, these numbers  should be by randomised group | Page 11 |
| Outcomes and estimation | 17 | For each objective, results including expressions of uncertainty (such as 95% confidence interval) for any  estimates. If relevant, these results should be by randomised group | Page 11-12 |
| Ancillary analyses | 18 | Results of any other analyses performed that could be used to inform the future definitive trial | NA |
| Harms | 19 | All important harms or unintended effects in each group (for specific guidance see CONSORT for harms) | Page 10 |
|  | 19a | If relevant, other important unintended consequences | NA |
| Discussion | | | |
| Limitations | 20 | Pilot trial limitations, addressing sources of potential bias and remaining uncertainty about feasibility | Page 15 |
| Generalisability | 21 | Generalisability (applicability) of pilot trial methods and findings to future definitive trial and other studies | Page 13-15 |
| Interpretation | 22 | Interpretation consistent with pilot trial objectives and findings, balancing potential benefits and harms, and  considering other relevant evidence | Page 13-15 |
|  | 22a | Implications for progression from pilot to future definitive trial, including any proposed amendments | Page 14-15 |
| Other information | | |  |
| Registration | 23 | Registration number for pilot trial and name of trial registry | Page 2,5 |
| Protocol | 24 | Where the pilot trial protocol can be accessed, if available | Page 5 |
| Funding | 25 | Sources of funding and other support (such as supply of drugs), role of funders | Page 19 |
|  | 26 | Ethical approval or approval by research review committee, confirmed with reference number | Page 5 |

Table S2. Facilitator adherence to OHP-CHR intervention components

| **Components/Key elements** | **Number of Applicable/n** | **Percentage of delivered (% of number of applicable)** |
| --- | --- | --- |
| **Session one Introduction to OHP**  **What is Optimal Health?** | | |
| 1.1 Introductions and acknowledgements.  Overview of wellbeing approach to managing self. Explore groups expectations and experiences of wellbeing | 29 | 29 (100%) |
| 1.2 What is wellbeing?  What is wellbeing management?  Where am I at today with my wellbeing?  What are the positive and negatives we inherently do that impacts our health? | 29 | 29 (100%) |
| Exploring domains of wellbeing  1.3 What’s our understanding of the six domains of wellbeing?  Physical, emotional, social, spiritual (values), occupational (engagement), intellectual.  Establishing one’s own satisfaction with domains of wellbeing.  How does our wellbeing impact me? | 29 | 29 (100%) |
| **Session Two**  **I-Can-Do Model Part One**  **Strengths and Vulnerabilities** | | |
| 2.1 Exploring strengths that have an impact on wellbeing.  Identify key strengths – personal qualities and environmental strengths, skill and talents, and interests and aspirations. | 28 | 28(100%) |
| 2.2 Understanding personal vulnerabilities that impact on wellbeing.  Identify key vulnerabilities - personal qualities and genetic factors as well as social determinants of heath. | 28 | 28 (100%) |
| 2.3 Discovering the relevance of the Health Plans when discussing strengths and vulnerabilities.  Health Plan 1 containing what one currently does to maintain wellbeing. | 28 | 27 (96.4%) |
| 2.4 The I Can Do Model or self-efficacy is a key concept of OHP Wellbeing to develop and maintain health plans. | 28 | 28 (100%) |
| **Session Three**  **I-Can-Do Model Part Two**  **Stressors and Strategies** | | |
| 3.1 Understanding personal stressors that impact on wellbeing. | 28 | 29 (100%) |
| 3.2 Positive and negative stressors and what happens in the mind and body.  Explore impacts of cumulative stress, stress thresholds and identifying cycles of crisis. | 28 | 29 (100%) |
| 3.3 Understanding personal strategies that impact on wellbeing.  Identify stressors and current strategies and their effectiveness.  Lowering the stress threshold to move away from crisis points. | 28 | 28 (100%) |
| 3.4 Understand the relevance of the Health Plans when discussing stressors and strategies. | 28 | 27 (96.4%) |
| 3.5 Important to notice early warning signs and support is required while maintaining self-agency. | 28 | 27 (96.4%) |
| **Session Four**  **Medication and Metabolic Monitoring** | | |
| 4.1 Exploring the importance of good physical health. | 28 | 25 (89.3%) |
| 4.2 Reinforcing a healthy lifestyle balance with nutrition, rest and exercise.  Improve overall health literacy | 28 | 24 (85.7%) |
| 4.3 Deepen collaboration with clinical services, shared decision making | 28 | 24 (85.7%) |
| **Session Five**  **Collaborative Partners and Strategies** | | |
| 5.1 Exploring communication styles. | 28 | 22 (78.6%) |
| 5.2 Exploring emotional intelligence.  Defining communication style alignment.  Identify current collaborative partners and networks of support. | 28 | 22 (78.6%) |
| 5.3 Importance of collaboration and support network.  Identify health strategies linked with key partnerships.  Establish strategies for optimal health supported by key partnerships | 28 | 28 (100%) |
| 5.4 Understand the relevance of the Health Plans when discussing episode of illness.  Health Plan 3 focuses on developing strategies to reduce the risk of an episode of illness as well as during episode of illness to maintain self-determination.  Revise Health Plans 1 and 2 and build a safety plan, documenting individual needs. | 28 | 25(89.3%) |
| **Session Six**  **Visioning and Goal Setting Part One**  **Defining Change** | | |
| 6.1 Identify past events and strategies and their impact on health.  Value of mapping experience in relation to level of wellbeing gaining insight into past events and their impact on health. | 26 | 25 (96.2%) |
| 6.2 Can use the Timeline Activity tool for future planning.  Revisit the health wheel to identify areas of health that need to improve or change. | 26 | 23 (88.5%) |
| 6.3 Explore the participant’s wellbeing and identify gaps that need addressing.  Use decisional balance if ambivalence exists.  Define the change and the importance and confidence in reaching that goal/objective/outcome. | 26 | 23 (88.5%) |
| 6.4 Motivation is influence by perceived:   - Competence - Autonomy - Relatedness | 26 | 24 (92.3%) |
| **Session Seven**  **Visioning and Goal Setting Part Two**  **Creative Problem Solving and Goal Setting** | | |
| 7.1 The steps in creative problem solving include:   - realise there is a problem, - define the problem, - brainstorm possible solutions, - evaluate all possible solutions, - select one solution, - plan the solution, - implement the plan; - then review the outcome. | 26 | 22 (84.6%) |
| 7.2 Support the participants’ self-agency to develop wellbeing leadership for self.  Share the principles of SMARTER or SMARTEST goals and importance of recognizing learning, acknowledging achievement and celebrating success.  Develop an attitude of accepting failure as essential feedback and learning.  Explore the growth or optimistic mindset. | 26 | 25 (96.2%) |
| 7.3 Goal setting principles include:  1. AAA rated goals: autonomous, approach, achievable  2. Approach goals versus avoidance goals  3. Proximal or Distal goals  4. Stretch Goal  5. Maximum of 3 goals  6. Duration of 12 weeks or less  7. 70% to 80% confidence | 26 | 21 (80.8%) |
| **Session Eight**  **Building Health Plans**  **My health Journal** | | |
| 8.1 Revisit Timeline activity  Value of mapping experience in relation to level of well-being gaining insight into past events and their impacts on health. | 26 | 23 (88.5%) |
| 8.2 Understand Health Plan 1 as a daily health plan,  Health Plan 2 as a strategy for suboptimal health and  Health Plan 3 as an action plan for an episode of illness.  Health Plans 1, 2 & 3 can be used as advanced care plan. | 26 | 25 (96.2%) |
| 8.3 Introduce the Health Journal as a way to stay connected with the principal processes of the program. | 26 | 13 (50.0%) |
| 8.4 Articulating the learning and identifying strategies for self beyond the workshop.  Identify the positive impacts of self-managing one’s own wellbeing. | 26 | 25 (96.2%) |

Table S3. Comparisons between participants with follow-up and retained in study and those who were not included in final exploratory analyses

| **Variables** | **Retained**  **n=26** | **Withdrawn**  **n=4** | **Test Statistic** | |
| --- | --- | --- | --- | --- |
|  | Mean (SD) | Mean (SD) | **t** | **P value** |
| Age in years | 22.0 (3.7) | 21.3 (4.0) | 0.35 | .726 |
| SOPS positive | 11.0 (4.2) | 11.0 (4.5) | -0.02 | .987 |
| SOPS negative | 10.9 (5.1) | 9.0 (4.5) | 0.72 | .480 |
| GF:R | 5.73 (1.8) | 7.0 (2.0) | -1.16 | .257 |
| GF:S | 6.4 (1.4) | 7.7 (1.5) | -1.41 | .171 |
| CD-RISC | 45.1 (16.1) | 53.7 (8.0) | -0.90 | .376 |
| CDSS | 5.7 (4.2) | 3.3 2.5) | 0.95 | .350 |
| STAI-S | 55.4 (11.7) | 46.7 (1.5) | 1.27 | .214 |
| STAI-T | 61.4 (10.4) | 52.0 (7.1) | 1.25 | .224 |
|  | **n (%)** | **n (%)** | **P value** | |
| Gender identity |  |  | 0.544 | |
| Man | 9 (33.3%) | 1 (25%) |
| Woman | 13 (50.0%) | 2 (50%) |
| Non-binary | 3 (13.3%) | 1 (25%) |
| Ethnicity background |  |  | 0.648 | |
| White | 12 (46.2%) | 2 (50%) |
| Others | 14 (53.8%) | 2 (50%) |
| Relationship status |  |  | 0.563 | |
| Single | 18 (69.2%) | 3 (75%) |
| In a relationship | 8 (30.7%) | 1 (25%) |
| Living arrangement |  |  | 0.169 | |
| Living with family/partner | 22 (84.6%) | 2 (50%) |
| Living on own/roommate | 4 (15.4%) | 2 (50%) |
| Current employment |  |  | 0.716 | |
| Student or employment | 18 (69.2%) | 3 (75%) |
| Not working/not in school | 8 (30.7%) | 1 (25%) |
